# Analysis of the plant hormone expression profile during somatic embryogenesis induction in teak (*Tectona grandis*)

**DOI:** 10.3389/fpls.2024.1429575

**Published:** 2024-10-07

**Authors:** Wenlong Zhou, Guang Yang, Dongkang Pan, Xianbang Wang, Qiang Han, Yaqi Qin, Kunliang Li, Guihua Huang

**Affiliations:** ^1^ Research Institute of Tropical Forestry, Chinese Academy of Forestry, Guangzhou, China; ^2^ Nanjing Forestry University, Nanjing, China; ^3^ Guangdong Eco-engineering Polytechnic, Guangzhou, China

**Keywords:** gene regulation, hormone profiling, somatic embryogenesis, *Tectona grandis*, transcriptomics

## Abstract

Plant somatic embryogenesis (SE) is an efficient regeneration system for propagation. It involves the regulation of a complex molecular regulatory network encompassing endogenous hormone synthesis, metabolism, and signal transduction processes, induced through exogenous plant growth regulators. Previous studies have focused primarily on traditional propagation methods for *Tectona grandis*, but there is limited knowledge on SE and its hormonal regulatory mechanisms. In our study, different SE stages, including the nonembryogenic callus (NEC), embryogenic callus (EC), and globular and heart-shaped embryo (E-SEs) stages, were induced in teak cotyledons incubated on MS medium supplemented with 0.1 mg/L thidiazuron (TDZ). Morphological and histological observations indicated that EC primarily originates from the development of embryogenic cell clusters. During SE induction, the levels of six classes of endogenous hormones, IAA, CTK, ETH, ABA, SA, and JA, changed significantly. Transcriptome analysis revealed that endogenous hormones participate in SE induction in teak through various biological processes, such as biosynthesis, metabolism, and signal transduction pathways. We found that IAA biosynthesis primarily occurs through the IAM pathway during these three stages. The ETH receptor kinase gene *SERF1* exhibited the highest expression levels in E-SEs. The ABA-, SA-, and JA-related signal transduction genes *ABI3*, *NPR1*, and *JAZ* exhibited no differential expression during different stages. Moreover, key encoding genes of SE regulators, including *WUS*, *WOX* and *SERK*, were differentially expressed during SE. In conclusion, this study offers insights into the roles of endogenous hormones and their interactions during SE induction.

## 1 Introduction

Teak (*Tectona grandis* L. f.) belongs to the Verbenaceae family and is a large deciduous tree species known for its elegant woodgrain, hardy wood, and durability. It is widely used in furniture manufacturing, decorative architecture, shipbuilding, and musical instrument production due to its qualities, demonstrating significant economic value (Tewari, 1992; Liang et al., 2020). Moreover, the ecological importance of teak cannot be overlooked. Its dense forests not only provide rich habitat and oxygen but also play a crucial role in soil and water conservation, as well as in ecological balance, making teak essential species for regional ecological environments and biodiversity conservation (Yasodha et al., 2018; Behera et al., 2023). Research on propagation techniques is particularly important for the effective utilization and conservation of teak resources. Traditional teak propagation methods, including via seed, cuttings, and tissue culture, are the main rapid propagation techniques for shoot proliferation (Chen et al., 2013; Li et al., 2017). However, there are drawbacks to seed reproduction and propagation with cuttings, such as long cycles and low success rates. Tissue culture techniques come with challenges such as low multiplication and rooting rates (Wu et al., 2017; Liao et al., 2017). Therefore, establishing efficient regeneration systems, including with somatic embryogenesis (SE), is urgently needed.

SE is characterized by rapid development, a high proliferation rate, relatively genetic stability, and good uniformity in regenerated individuals (Gulzar et al., 2020). *In vitro* SE represents a prospective approach for mass production, preservation of genetic resources, and improvement of genetic traits. SE is an *in vitro* regeneration process in which somatic cells are induced to produce somatic embryos that further develop into entire plants. This process undergoes a comprehensive process analogous to zygotic embryonic development (Von Arnold et al., 2002). SE in plants can be classified into two primary types: direct SE (DSE) and indirect SE (ISE). In DSE, the explant directly initiates the formation of somatic embryos, thereby circumventing the callus phase and leading to the propagation of mature plants, while ISE begins with the induction of callus, which can be either embryogenic callus (EC) or non-embryogenic callus (NEC) (Méndez-Hernández et al., 2019; Wen et al., 2020). Moreover, NEC can further differentiate into embryogenic callus (Qi et al., 2021). The formation of callus tissue is generally considered a manifestation of dedifferentiated cell status. EC continues to differentiate, forming globular embryos (GEs), heart-shaped embryos (HEs), torpedo-shaped embryos (TEs), and cotyledonary embryos (CEs), ultimately developing into mature embryos (Zhao et al., 2017). Both types of embryogenesis have been reported in various plant species, such as *Zea mays L.* (Ding, 2017), *Oryza sativa* (Indoliya et al., 2016), *Triticum aestivum* (Chu et al., 2017), *Liquidambar styraciflua* (Qi et al., 2021), *Arabidopsis thaliana* (Gliwicka et al., 2013), and *Hippeastrum* ‘Bangkok Rose’ (Zeng et al., 2023).

Hormones, as plant growth regulators, play a significant role in SE induction. The application of exogenous hormones can lead to changes in the metabolic levels of endogenous hormones, thereby inducing somatic cells to acquire embryogenic competence (Yang and Zhang, 2010). The auxin-like herbicide 2,4- dichlorophenoxyacetic acid (2,4-D) can promote the cell growth and increase the concentration of endogenous indole-3-acetic acid (IAA) (Garcia et al., 2019; Ding, 2017). The cytokinin-like plant growth regulator thidiazuron (TDZ) has the dual effects of auxin and cytokinin, promoting plant shoot regeneration and reproduction, enhancing callus growth, and inducing SE (Paul et al., 2011). TDZ treatments have been found to induce both ISE and DSE in *Murraya* and *Tolumnia* Louise Elmore, respectively (Paul et al., 2011; Shen et al., 2018). In addition to TDZ-mediated plant SE, extensive research suggests that endogenous hormone synthesis, metabolism, and signal transduction are instrumental in the proliferation of meristematic tissue cells, the transformation of somatic cells into embryogenic tissues, and the subsequent differentiation and maturation of somatic embryos (Haque and Ghosh, 2017; Méndez-Hernández et al., 2019). The indole-3-acetamide (IAM), indole-3-pyruvic acid (IPA), tryptamine (TAM), and indole-3-acetaldoxime (IAOX) pathways are the main pathways in tryptophan-dependent IAA biosynthesis (Mano and Nemoto, 2012). Moreover, in *Arabidopsis*, YUCCA (*YUC*) -mediated local IAA biosynthesis is required for SE induction (Bai, 2013). Many embryogenic systems exhibit an instantaneous increase in endogenous IAA concentration, with explants possessing high levels of endogenous auxin being more easily induced to dedifferentiate (Jiménez, 2005). Cytokinin (CTK) interacts with auxin during plant SE. In Arabidopsis, the response regulator ARR1, which is a transcription factor for genes immediately responsive to cytokinins, can enhance callus formation in media supplemented with auxin when overexpressed (Sakai et al., 2001; Hwang et al., 2012). Abscisic acid (ABA) is essential in *Arabidopsis* SE (Bai, 2013), and the acquisition of cell embryogenic capacity is severely impaired by strong inhibitory treatment with the ABA biosynthesis inhibitor fluridone (Su et al., 2013). Mutations in the ABA biosynthesis gene *ABA2* also affect embryogenic induction capacity. The constitutive ethylene (ETH) signaling response can inhibit the synthesis of local auxin via the IPA pathway mediated by the *YUC* gene family, thereby inhibiting somatic embryo development (Bai, 2013). Treatment of cotton with a certain concentration of jasmonate (JA) results in the production of numerous secondary somatic embryos on the surface of globular embryos (Ge, 2016). Salicylic acid (SA) can induce SE in mature *Panax quinquefolium* seed embryos (Liu et al., 2016). However, in teak, the endogenous hormone regulatory mechanisms during SE remain unclear.

Many studies have shown that ectopic overexpression of embryonic or meristem regulatory factors can promote cell dedifferentiation, thereby facilitating SE in various species. The identification of key genes in model plants could elucidate the mechanisms underlying SE. Notably, the overexpression of several key regulators, including the *SOMATIC EMBRYOGENESIS RECEPTOR-LIKE KINASE 1* gene (*SERK1*), increases the efficiency of somatic cell initiation in *Arabidopsis* (Hecht et al., 2001; Rupps et al., 2016). *BABY BOOM* (*BBM*) encodes an APETALA2 (AP2) domain transcription factor that is preferentially expressed in developing embryos and seeds (Ding, 2017). Overexpression of *BBM* can trigger spontaneous formation of somatic embryos (Maulidiya et al., 2020). The homologous domain transcription factor (WUS), which can regulate the formation and maintenance of stem cells, is an early marker gene for shoot apical meristem (SAM) initiation in embryos (Elhiti et al., 2010). *LEAFY COTYLEDON2* (*LEC2*) (Rupps et al., 2016), *ABSCISIC ACID INSENSITIVE3* (*ABI3*), and *FUSCA3* (*FUS3*), which encode B3 domain transcription factors that are key regulators of embryogenesis (Freitas et al., 2019), have been identified and verified as direct target genes of *AGAMOUS-LIKE15* (AGL15). In addition to these genes, *WUSCHEL HOMEOBOX 2* (*WOX2*) also plays key roles in SE (Tvorogova et al., 2015). However, in teak, the expression changes and regulatory mechanisms of these genes during SE remain unknown.

A comprehensive study of the molecular regulatory mechanisms involved in teak SE is crucial for the establishment of efficient regenerative systems. In this study, non-embryogenic callus (NEC), embryogenic callus (EC), and globular and heart-shaped embryos were induced in teak by the addition of the exogenous hormone TDZ. Different SE stages were subjected to endogenous hormone measurement and RNA-seq to elucidate the correlation between plant endogenous hormone levels and the synthesis, metabolism, and signal transduction of these hormones. These findings contribute to the establishment of an effective regeneration system and provide valuable insights into the molecular regulation of SE in teak.

## 2 Materials and methods

### 2.1 Plant material and sample collection

The present study utilized fully developed teak seeds from the same genetic lineage. The seeds were subjected to 5 minutes of mercuric chloride disinfection, followed by five aseptic double-distilled water rinses, and then incubated onto Murashige and Skoog (MS) medium and 3% (w/v) sucrose and 0.8% (w/v) agar (pH 6) for seed germination (Akram and Aftab, 2016). Upon germination and growth of the seed to the stage of two cotyledons fully expanding, the cotyledons are excised and transferred to a modified MS culture medium supplemented with 0.1 mg/L TDZ (dissolved in Dimethyl Sulfoxide, Sangon Biotech, Shanghai, China) for tissue culture. Cotyledons are subcultured once a month in the medium until the NEC morphological characteristics are observed, at which point they are transferred for the next stage of induction under the same tissue-culture condition as mentioned above. During the stage where NEC are induced to become EC, subcultures are conducted every three weeks. When EC morphological characteristics emerge, they are transferred to the same culture medium condition for subsequent stage cultivation. During the stage where EC are induced to form SE, subcultures are again conducted every three weeks. When globular and heart-shaped embryonic morphological characteristics appear, we proceed with sample collection. All the cultures were maintained at 26°C and 50%–70% humidity under a 16 h light/8 h dark photoperiod. For tissues with similar morphological structural characteristics at the same stage, including NEC, EC, and early somatic embryos (E-SEs, encompassing both globular and heart-shaped embryos), were selected. The samples were promptly frozen in liquid nitrogen and stored in a -80 °C ultralow temperature freezer for subsequent transcriptome sequencing (RNA-seq), endogenous hormone analysis, and reverse transcription-quantitative polymerase chain reaction (RT-qPCR).

### 2.2 Morphological and histological observations

During the process of SE induction, samples at different stages were collected for morphological and histological observation. Morphological observations were conducted and documented using a dissecting microscope (Zeiss Stemi 508, Germany). Germany). For histological observations, the samples were fixed in FAA solution for 24 hours, dehydrated in a series of alcohol gradients, subjected to transparency treatment (immersed in xylene for approximately 30-50 minutes), subjected to paraffin embedding, and sectioned with a rotary microtome at a thickness of approximately 8 μm–10 μm. Staining was performed using the Safranin-O and Fast Green double staining method following the previously described protocol (Zhang et al., 2021). Subsequently, tissue sections at various stages were observed using an optical microscope (Zeiss Primostar 3, Germany), and morphological observations were documented by capturing images with a camera (SONY, Japan).

### 2.3 Assessment of endogenous hormones during different stages of SE induction in teak

Samples from different tissue parts stored at -80°C were retrieved and pulverized into powder using a grinding method at 30 Hz for 1 minute. Fifty milligrams of plant sample was weighed into a 2 mL plastic microtube, frozen in liquid nitrogen, and dissolved in 1 mL of methanol/water/formic acid (15:4:1, v/v/v). Ten microliters of an internal standard mixed solution (100 ng/mL) was added to the extract as an internal standard (IS) for quantification. The mixture was vortexed for 10 min and then centrifuged for 5 min (12000 r/min at 4°C). The supernatant was transferred to clean plastic microtubes, evaporated to dryness, dissolved in 100 μL of 80% methanol (v/v), and filtered through a 0.22 μm membrane filter for further LC−MS/MS analysis. All sample extracts were analyzed using a UPLC-ESI-MS/MS system (UPLC, ExionLC™ AD, https://sciex.com.cn/, MS, QTRAP^®^ 6500+, https://sciex.com.cn/). Quantitative signal intensity data corresponding to various concentration standards were obtained. A standard curve for different substances was constructed by plotting the concentration ratio of external standard to internal standard against the area ratio of external standard to internal standard. The integrated peak area ratio values of all detected samples were then substituted into the linear equation of the standard curve for calculation. Subsequently, upon further substitution into the calculation formula, the actual content of the substance in the samples was determined. Hormone content (ng/g) = c*V/1000/m. C (ng/ml) represents the concentration values obtained by substituting the integrated peak area ratio from the samples into the standard curve. V denotes the volume of the solution used for reconstitution (μl), and m represents the mass of the sampled material (g).

### 2.4 RNA-seq analysis

Thirty samples from three induced SE stages in teak were grouped for processing. Each stage comprised three sample groups. Total RNA extraction was performed using the RNAprep Pure Polysaccharide Polyphenol Plant Total RNA Extraction Kit (TIANGEN, Beijing, China) on the nine sample groups from the three stages. RNA quality and integrity were assessed using 1% agarose gel electrophoresis and an Agilent 2100 Bioanalyzer (Agilent America). Nine high-quality RNA samples were selected for further analysis, with each sample containing 100 ng RNA. Subsequent experiments, including RNA-Seq library construction, sequencing, and initial data processing for each replicate sample, were conducted by MetWare Biotechnology Inc. (MetWare, Wuhan, China) using Illumina HiSeq sequencing technology. Moreover, PCA analysis in R software (www.r-project.org/) (version V3.5.1) was conducted using the built-in statistical function prcomp. The prcomp function was configured with the parameter scale=True, indicating unit variance scaling of the data, and based on the calculation of Euclidean distances between samples.

### 2.5 Differential gene expression analyses and functional annotation

To assess differential expression levels during different stages, transcript reads were counted using featureCounts (Liao et al., 2014), and analysis of differentially expressed genes (DEGs) was conducted using the DESeq2 R package (Varet et al., 2016). The expression level of each transcript was calculated according to the fragments per kilobase of exon per million mapped reads (FPKM) method. DEGs were defined according to the thresholds of |log2 Fold Change (FC)| ≥ 1 and adjusted false discovery rate (FDR) < 0.05, with the Benjamini−Hochberg method applied for FDR control (Dubitzky et al., 2013). Genes hierarchical clustering analysis heatmap was generated using TBtools (Chen et al., 2020).

Gene functions were annotated based on the nonredundant (nr) (Bleasby and Wootton, 1990), Pfam (Finn et al., 2003), EuKaryotic Orthologous Groups (KOG) (Wang et al., 2018), Clusters of Orthologous Genes (COG) (Meereis and Kaufmann., 2008), Non-supervised Orthologous Groups (eggNOG) (Muller et al., 2010), Swiss-Prot (Bairoch and Apweiler, 1999), Kyoto Encyclopedia of Genes and Genomes (KEGG) (Ogata et al., 1999), and Gene Ontology (GO) databases (Gene Ontology Consortium, 2001). GO and KEGG functional enrichment analyses of DEGs were performed to identify which GO terms or KEGG pathways were significantly enriched in DEGs. GO terms with corrected P values ≤0.05 were considered significantly enriched terms. KEGG enrichment was determined by the Rich factor, Q-value, and number of enriched genes in this pathway. A Q value ≤0.05 was defined as a gene that showed significant differential expression.

### 2.6 Real-time quantitative PCR

We selected 12 DEGs to validate the RNA-Seq data using RT-qPCR. Total RNA extraction was performed in the same manner as for RNA-seq. A HiScript^®^ III 1st Strand cDNA Synthesis Kit (Vazyme, Nanjing, China) was used for first-strand cDNA synthesis, and approximately 1 µg of total RNA was used for reverse transcription. RT-qPCR primers (**Supplementary Table 1**) (Tsingke Biotech Co., Ltd, Beijing, China) were designed using Primer 3plus (https://www.primer3plus.com/), with the elongation factor TgEf-1α chosen as the reference gene for normalization (Galeano et al., 2014). Primer specificity was validated by RT-qPCR melt curve analysis and agarose gel electrophoresis for specific band confirmation. qPCR was performed using a Roche/Light Cycler 480II (Roche, Switzerland) detection system and a Taq Pro Universal SYBR qPCR Master Mix kit (Vazyme, Nanjing, China) following the manufacturer’s instructions. A total of 2ul cDNA was added to the 20ul system for RT-qPCR. The relative expression levels of teak SE-related genes were calculated using the 2^−ΔΔCt^ method (Livak and Schmittgen, 2001). The data are presented as the mean ± standard deviation (SD), and all experiments were conducted in triplicate.

### 2.7 Statistical analyses

Statistically significant differences (P < 0.05) were determined by one-way ANOVA with Tukey’s test in IBM SPSS Statistics 26. All data were expressed as means ± standard deviation (SD). Samples were collected with three biological replicates each.

## 3 Results

### 3.1 Morphological and histological observation of somatic embryo in teak

**Figure 1:**
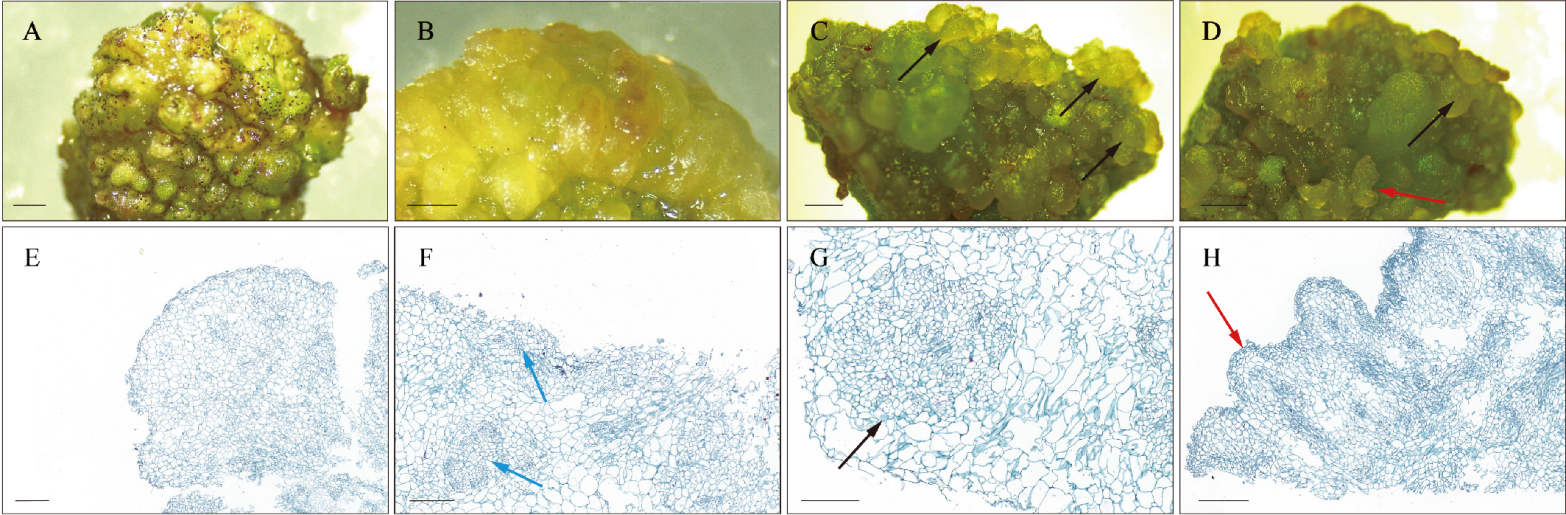
Morphology and histology during SE induction. **(A, E)** Non-embryogenic callus. **(B, F)** Embryogenic callus. **(C, D, G, H)** Early somatic embryos. The blue arrows indicate embryogenic cell clusters, the black arrows indicate globular embryos, and the red arrows indicate heart-shaped embryos. Histological staining employs the Safranin-O and Fast Green double staining method. Scale bar: **(A, C)** 3 mm; **(B, D)** 2 mm; **(E–H)** 200 μm.

In this study, we transferred cotyledons of teak germinated from seeds onto MS culture medium supplemented with an additional 0.1 mg/L TDZ for SE induction. Morphological changes during SE induction were observed. The initial development of NEC was characterized by green, dense and globular structure (**Figure 1A**). The NEC then underwent embryogenic transformation on TDZ-supplemented MS medium, resulting in light yellow embryogenic callus (**Figure 1B**). Subsequently, the embryogenic callus differentiated into translucent green globular embryos and heart-shaped embryos (**Figures 1C, D**). Histological observations of the samples were conducted using paraffin sections to assess the distinct NEC, EC, and E-SE tissue structures. The NEC exhibited larger cells, an irregular arrangement, and a lighter staining color (**Figure 1E**). Highly proliferative cells with a compact arrangement, forming embryogenic cell clusters exhibiting smaller cell size and intensified staining, are identified within the embryogenic callus, occurring either internally or on its surface. (**Figure 1F**). Moreover, GE and HE were found to occur at the periphery of embryogenic callus tissue (**Figures 1G, H**). These findings indicate that SE in teak occurs indirectly in MS medium supplemented with TDZ.

### 3.2 Analysis of transcriptome data at different SE stages induced in teak

**Figure 2:**
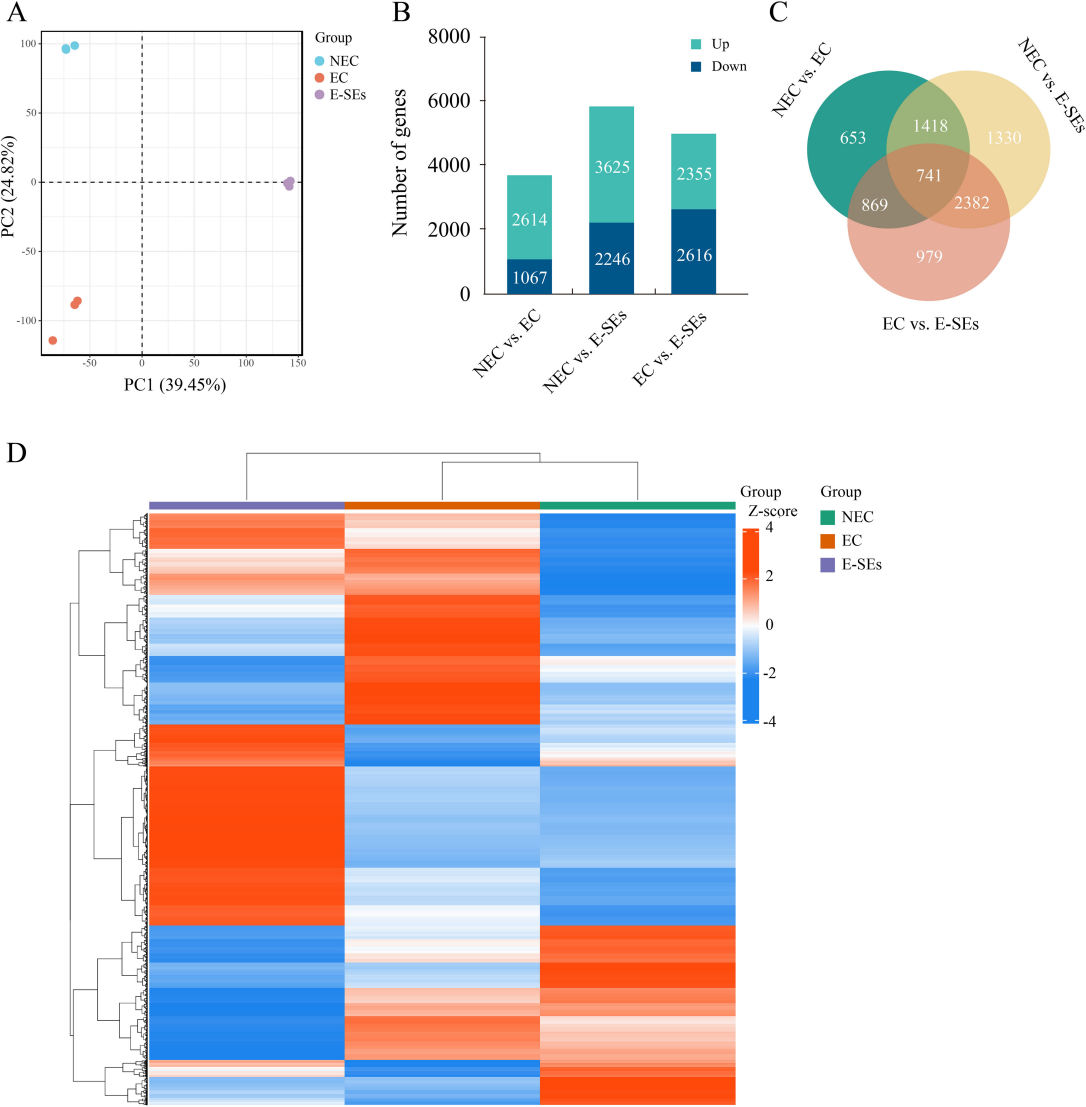
Analysis of transcriptome data. **(A)** PCA of the transcriptome profiles of NEC, EC and E-SEs. Each symbol represents one replicate. **(B)** Histogram illustrating the number of upregulated or downregulated DEGs in NEC vs. EC, EC vs. E-SEs, and NEC vs. E-SEs. **(C)** Venn diagram of all DEGs. **(D)** Hierarchical clustering analysis of DEGs at different SE stages induced in teak. Red indicates high relative gene expression, and green indicates low relative gene expression.

Transcriptome sequencing at the NEC, EC, and E-SEs stages was conducted using next-generation sequencing technology. A total of 42 million high-quality reads were obtained and aligned to the teak reference genome with HISAT2 (Kim et al., 2019; Zhao et al., 2019). An average of 89% of the reads were uniquely mapped (**Supplementary Table 2**) and used to calculate the normalized gene expression level as fragments per kilobase of FPKM. Furthermore, principal component analysis was performed on 9 samples from three tissue (**Figure 2A**). The results discrepancy between samples at different stages and reproducibility within each group.

To identify DEGs during various SE induced in teak, transcriptome data from three stages were analyzed using DESeq2 (Varet et al., 2016), with selection criteria of |log2 (FC)| ≥ 1 and FDR < 0.05. A total of 8372 genes were identified as DEGs during SE induction in teak (**Figures 2B, C**). Among these genes, the largest difference in gene expression was observed between NEC vs. E-SEs, with 5871 genes showing differential expression, of which 3625 genes were upregulated and 2246 genes were downregulated. The smallest number of DEGs was observed between NEC vs. EC, with 3681 differentially expressed genes, including 2614 upregulated and 1067 downregulated genes. There were 4971 genes differentially expressed between EC vs. E-SEs, with 2355 upregulated and 2616 downregulated genes (**Figures 2B, C**). The normalization of differential gene expression levels was conducted using the Z-score method (Cheadle et al., 2003), followed by gene-level hierarchical clustering analysis. The majority of DEGs exhibited the highest expression levels at the E-SEs stage, with a small subset showing the highest expression levels at the NEC stage (**Figure 2D**). The comparisons of the NEC vs. EC, EC vs. E-SEs, and NEC vs. E-SEs indicated that the DEGs were involved in SE induced by TDZ in teak.

### 3.3 GO enrichment analysis of DEGs in biological processes

**Figure 3:**
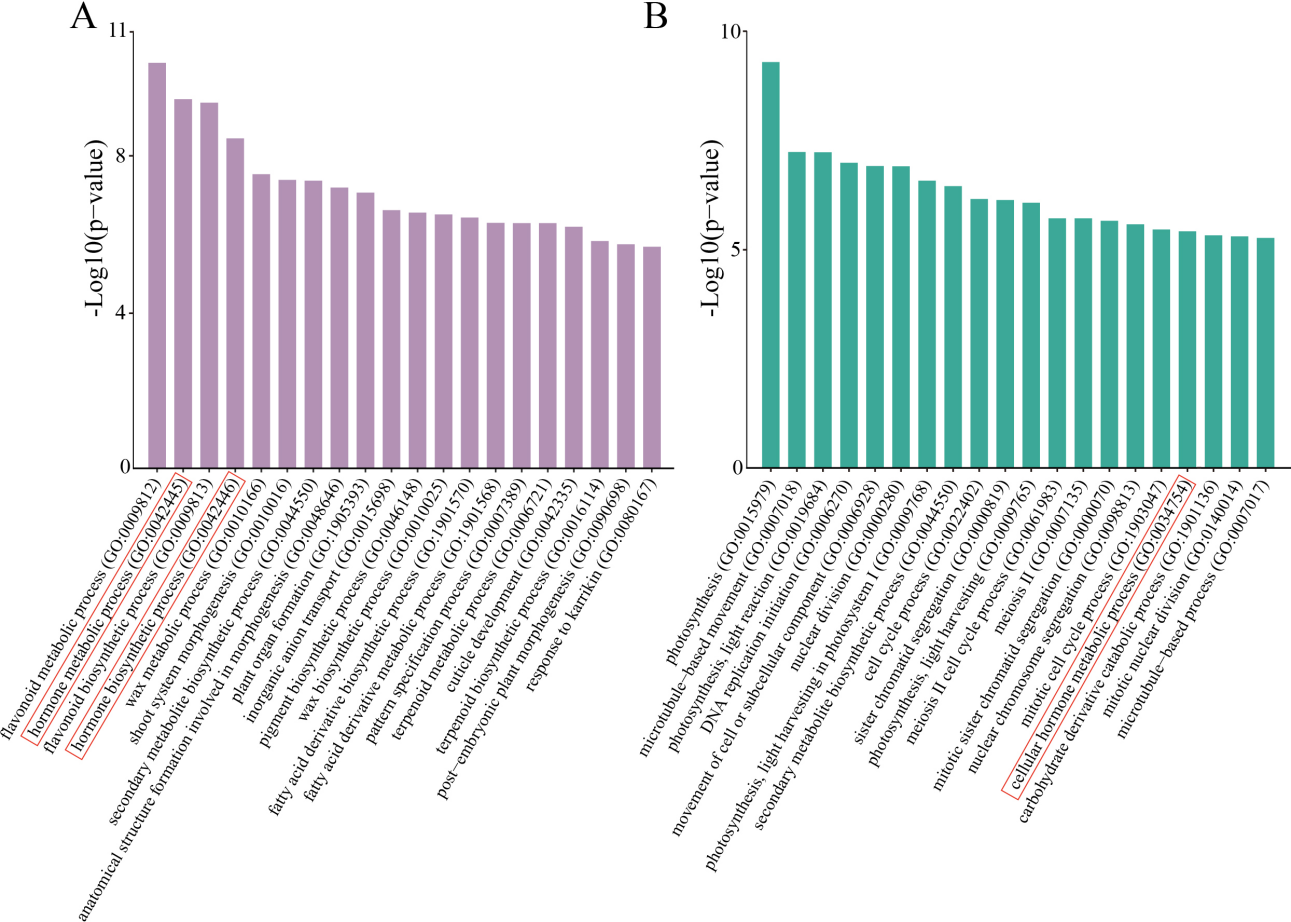
GO enrichment. **(A)** Top 20 enriched GO biological process terms in NEC vs. EC. **(B)** Top 20 enriched GO biological process terms in EC vs. E-SEs. The red frame denotes the biological processes associated with hormones.

To elucidate the biological processes underlying SE induction in teak, we performed GO term enrichment analysis of the DEGs in the NEC vs. EC and EC vs. E-SEs comparisons. Several biological processes, including hormone metabolic processes and secondary metabolite biosynthetic processes (GO: 0044550), were significantly enriched in both groups of DEGs (**Figure 3**). Hormone biosynthetic processes (GO: 0042446), flavonoid metabolism (GO: 0009812) and flavonoid synthesis (GO: 0009813) were significantly enriched in NEC vs. EC but not in EC vs. E-SEs (**Figure 3**), indicating that the DEGs involved in hormone biosynthesis predominantly function during the induction process of embryogenic callus tissue, while flavonoid metabolism and synthesis do not play a key role in somatic embryo development. Processes such as DNA replication initiation (GO: 0006270), cell cycle process (GO: 0022402), and sister chromatid segregation (GO: 0000819) were significantly enriched in EC vs. E-SEs, suggesting the involvement of cell division in embryogenic stage formation.

### 3.4 Phytohormone levels during SE induction in teak

**Figure 4:**
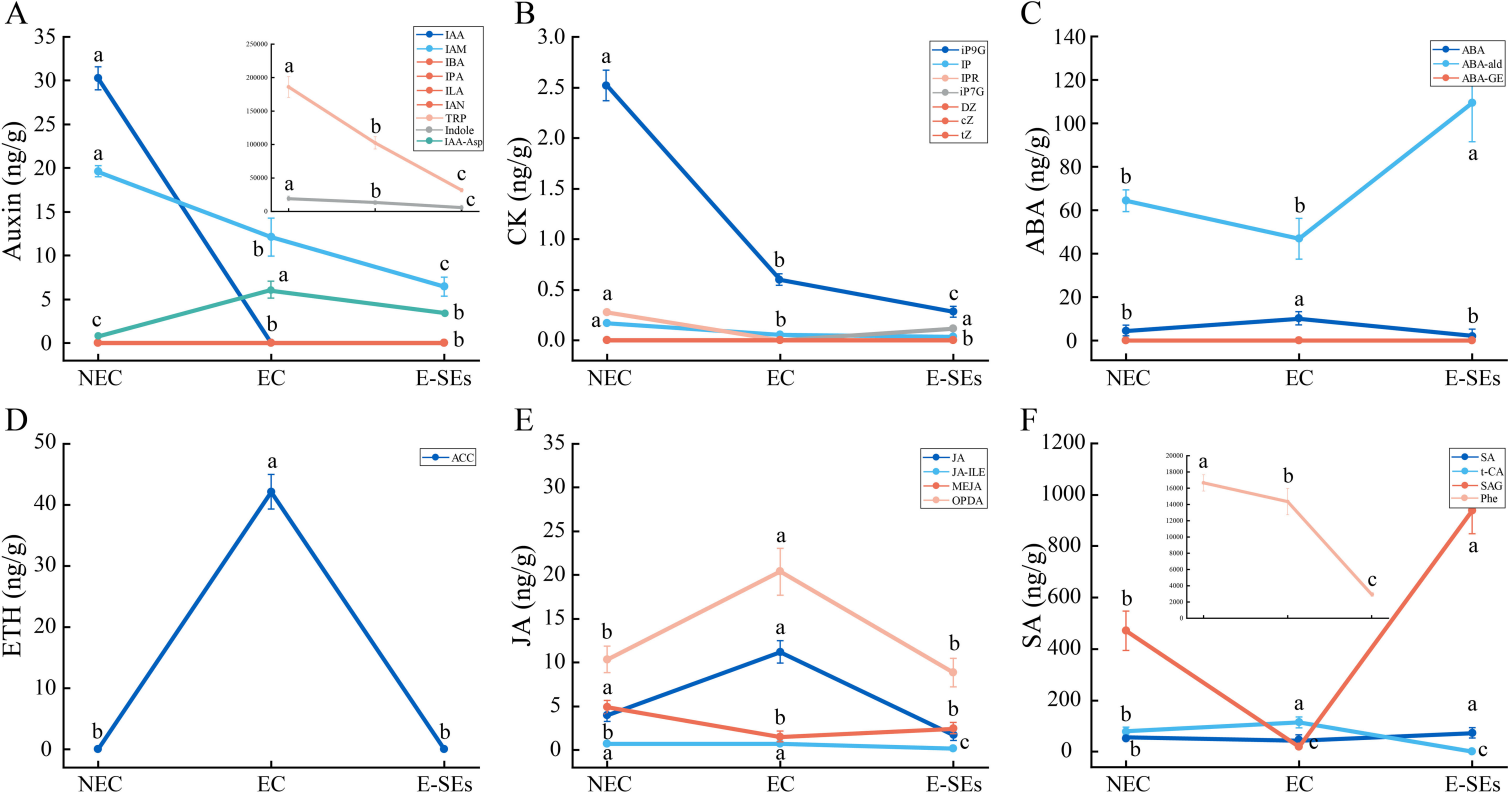
Dynamic changes in the levels of the hormones. **(A)** auxin, **(B)** CTK, **(C)** ABA, **(D)** ETH, **(E)** JA, and **(F)** SA at different stages. Data are shown as line graph with whiskers representing means ± SD. Statistically significant differences (P < 0.05) were determined by one-way ANOVA with Tukey’s test and were indicated with different letters.

Plant hormones play pivotal roles in plant growth and development. GO enrichment analysis of the DEGs revealed that plant hormone-related pathways were significantly enriched (**Figure 3**). To understand the role of plant hormones during SE induction in teak, we measured seven types of endogenous hormones—Auxin, CTK, ABA, ETH, JA, SA and gibberellic acid (GA), —across three developmental stages (**Figure 4**). The levels of the auxin-related substances tryptophan (TRP) and precursor indole, as well as the IAA, are significantly reduced in EC and E-SEs, and IAA is undetectable in EC and E-SEs (**Figure 4A**). Other auxin-related substances, such as indole-3-butyric acid (IBA), 3-indolepropionic acid (IPA), indole-3-lactic acid (ILA), and 3-indoleacetonitrile (IAN), were not detected during the three periods of teak somatic embryo development, indicating the crucial role of IAA during SE induction in teak. The levels of the cell division-related substances N6-isopentenyl-adenine-9-glucoside (iP9G), N6-isopentenyladenine (IP), and N6-isopentenyladenosine (iPR) decreased during SE induction (**Figure 4B**). ABA increased as NEC transitioned to EC but significantly decreased upon transition to E-SEs (**Figure 4C**). The levels of the ethylene precursors 1-aminocyclopropanecarboxylic acid (ACC), jasmonic acid-related JA, and cis(+)-12-oxophytodienoic acid (OPDA) significantly increased during the transition from NEC to EC and decreased significantly during the transition from EC to E-SEs, indicating their involvement in the transformation of teak EC to E-SEs under TDZ induction (**Figures 4D, E**). JA and SAG decreased as NEC transitioned to EC but significantly increased upon transition to E-SEs (**Figure 4F**). Additionally, the cytokinin substances trans-zeatin riboside (tZ), dihydrozeatin (DZ), and cis-zeatin (cZ) were not detected at any of the three stages, suggesting their minimal presence in the three tissue types (**Figure 4B**). However, further verification is needed to assess their involvement in teak SE induction. GA content was not detected during SE induction (**Supplementary Figure 1**).

### 3.5 Expression patterns of genes related to plant hormone synthesis and metabolism during SE induction in teak

Significant changes in hormone levels occurred during the SE induction in teak (**Figure 4**). To elucidate the underlying mechanisms of hormone action during SE induction, we integrated hormone expression profile data with transcriptome data and constructed six hormone biosynthetic and metabolic pathways based on previous studies (Mano and Nemoto, 2012; Urano et al., 2017; Kępczyńska and Orłowska, 2021). Through homologous alignment (**Supplementary Figures 2**-**7**), we identified homologous genes related to these processes (**Supplementary Table 3**) and generated an expression heatmap.

**Figure 5:**
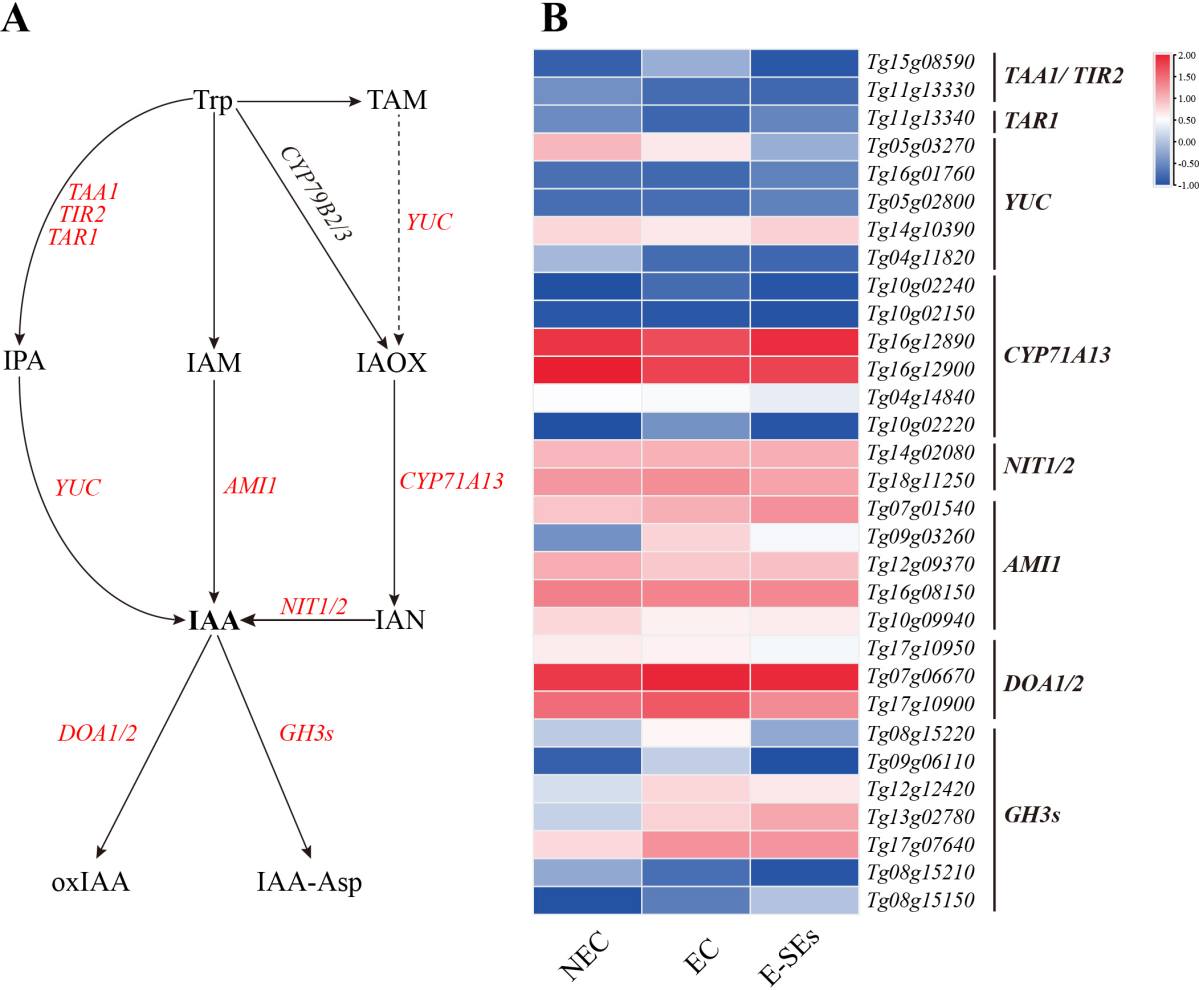
Dynamic changes in the expression of genes involved in IAA biosynthetic and metabolic pathways at different stages in teak. **(A)** Biosynthetic pathway of IAA. Substrates and products are denoted by plain text; genes encoding enzymes are denoted by italics. Red indicates the presence of homologous genes, black represents genes for which homology has not been detected. The details of the IAA biosynthetic and metabolic pathways were described in previous reports (Mano and Nemoto, 2012). **(B)** Expression of genes involved in the IAA biosynthetic and metabolic pathways at different stages. The heatmap shows the relative transcript levels of selected synthetic and metabolic genes. The color scale corresponds to the average log_10_ (FPKM+0.1). *TAA1*, tryptophan aminotransferase of *Arabidopsis* 1; *TAR1*, TAA-related 1; *TIR2*, tryptophan inhibitor response 2; *YUC*, YUCCA; *AMI1*, amidase 1; NIT, nitrilase; GH3s, gretchen hagen 3s; DOA, Auxin oxidase; CYP71A13, Cytochrome P450, Family 71.

Auxin plays a crucial role in plant SE (Nawy et al., 2008). IAA has been identified as the most potent hormone for SE induction. In our study, the key synthetic gene *AMI1* (*Tg07g01540*, *Tg09g03260*) of the IAM pathway showed differential expression during SE induction (>2-fold). Moreover, the expression level of the *Tg09g03260* gene peaked during the EC phase, while the *Tg07g01540* expression level continued to increase (**Figure 5**). The intermediate products IPA and IAN from the other three pathways were not detected at any of the three stages (**Figure 4A**). Genes involved in the biosynthesis and metabolism of IPA and IAN, including *TAA1*/*TAR1*/*TIR2* (three genes, *Tg15g08590*, *Tg11g13330*, and *Tg11g13340*), *YUC* (four genes, *Tg16g01760*, *Tg05g02800*, *Tg14g10390*, and *Tg04g11820*), CYP71A13 (six genes, *Tg10g02240*, *Tg10g02150*, *Tg16g12890*, *Tg16g12900*, *Tg04g14840*, and *Tg10g02220*), and *NIT1/2* (two genes, *Tg14g02080* and *Tg18g11250*), were found to be nondifferentially expressed genes or low transcript levels (FPKM < 1) (**Figure 5**). However, the expression levels of the other *YUC* (*Tg05g03270*) gradually decreased during SE induction (**Figure 5**). The deactivation of IAA was significant through the degradation pathways mediated by *DOA1/2* and *GH3s*, leading to the formation of 2-oxindole-3-acetic acid (oxIAA) and indole-3-acetyl-L-aspartic acid (IAA-Asp) (**Figure 5**). During SE induction, the expression levels of *GH3s* genes were increased compared to those during NEC, with four genes (*Tg08g15220*, *Tg09g06110*, *Tg12g12420*, and *Tg17g07640*) peaking in expression EC, showing a consistent trend with the changes in IAA-Asp levels, while the remaining *Tg13g02780* exhibited the highest expression in E-SEs. Furthermore, genes encoding enzymes involved in auxin metabolism pathways, *DAO1/2* (*Tg17g10900*), exhibited the highest expression at the EC stage (**Figure 5**). These findings indicate that the metabolic pathway plays a crucial role in the degradation of IAA during SE induction in teak.

**Figure 6:**
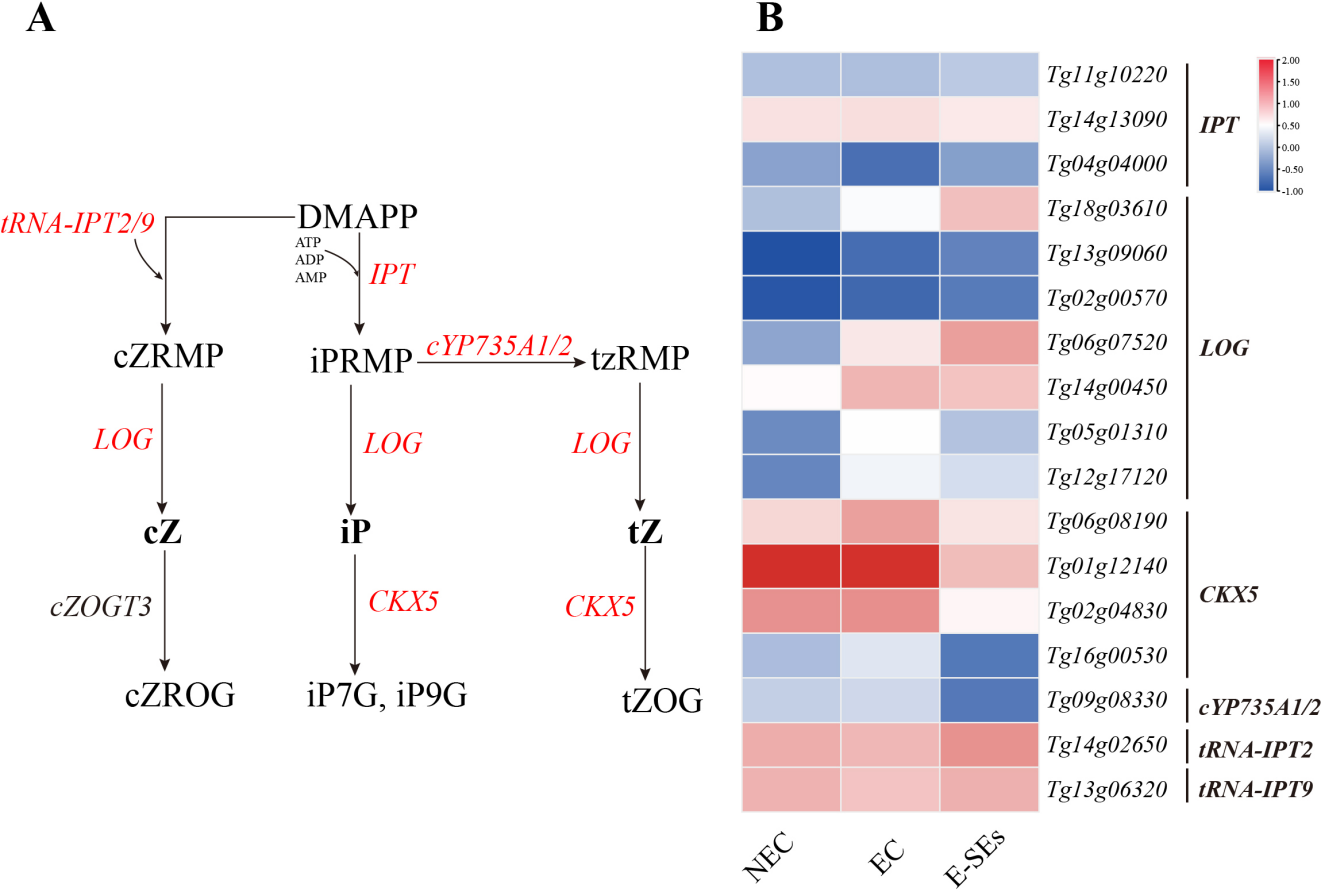
Dynamic changes in the expression of genes involved in CTK biosynthetic and metabolic pathways at different stages of SE in teak. **(A)** Biosynthetic pathway of CTK. Substrates and products are denoted by plain text; genes encoding enzymes are denoted by italics. Red indicates the presence of homologous genes, black represents genes for which homology has not been detected. The detailed CTK biosynthetic and metabolic pathways were described in previous reports (Hirose et al., 2007). **(B)** Expression of genes involved in the CTK biosynthetic and metabolic pathways at different stages of SE. The heatmap shows the relative transcript levels of selected synthetic and metabolic genes. The color scale corresponds to the average log_10_(FPKM+0.1). DAMPP, dimethylallyl diphosphate; cZRMP, cis-zeatin riboside monophosphate; iPRMP, N-6-iso-pentenyladenosine-5’-monophosphate; tzRMP, 9-Ribosyl-trans-zeatin 5’-monophosphate; cZROG, cis-zeatin-O-glucoside riboside; tZOG, trans-zeatin-O-glucoside; *tRNA-IPT*, Trnaisopentenyltransferase; *IPTs*, isopentenyltransferases; *cYP735A1,2*, Cytochrome P450; *LOG*, Lonely Guy; *CKX*, Cytokinin oxidase.

Cytokinins are known to play a role in the early globular embryo stage of *Arabidopsis thaliana* (Müller and Sheen, 2008). In our study, the key gene involved in IP synthesis, the *LOG*, showed increased expression levels of five differentially expressed genes (*Tg18g03610*, *Tg06g07520*, *Tg14g00450*, *Tg05g01310*, and *Tg12g17120*) during SE induction (>2-fold), with three genes (*Tg14g00450*, *Tg05g01310*, and *Tg12g17120*) peaking in expression during the EC stage (**Figure 6**). Analysis of cytokinin metabolism-related gene expression revealed increased expression of the cytokinin oxidase gene CK oxidase (*CKX5*) during the EC stages, except for in *Tg01g12140*. All CKX5 genes exhibited the lowest expression levels at the E-SEs stage (**Figure 6**). These results suggest that the levels of iP are regulated by *LOG* and *CKXs*. Furthermore, the endogenous hormones cZ were not detected at any of the three stages (**Figure 4**), and encoding the precursors of cZ synthesis genes, *tRNA-IPT2* (*Tg14g02650*) and *tRNA-IPT9* (*Tg13g06320*), did not show differential expression across the three induced SE stages. Additionally, encoding the precursors of tZ synthesis, *cYP735A1/2* (*Tg09g08330*) significantly decreased during the E-SEs phase.

**Figure 7:**
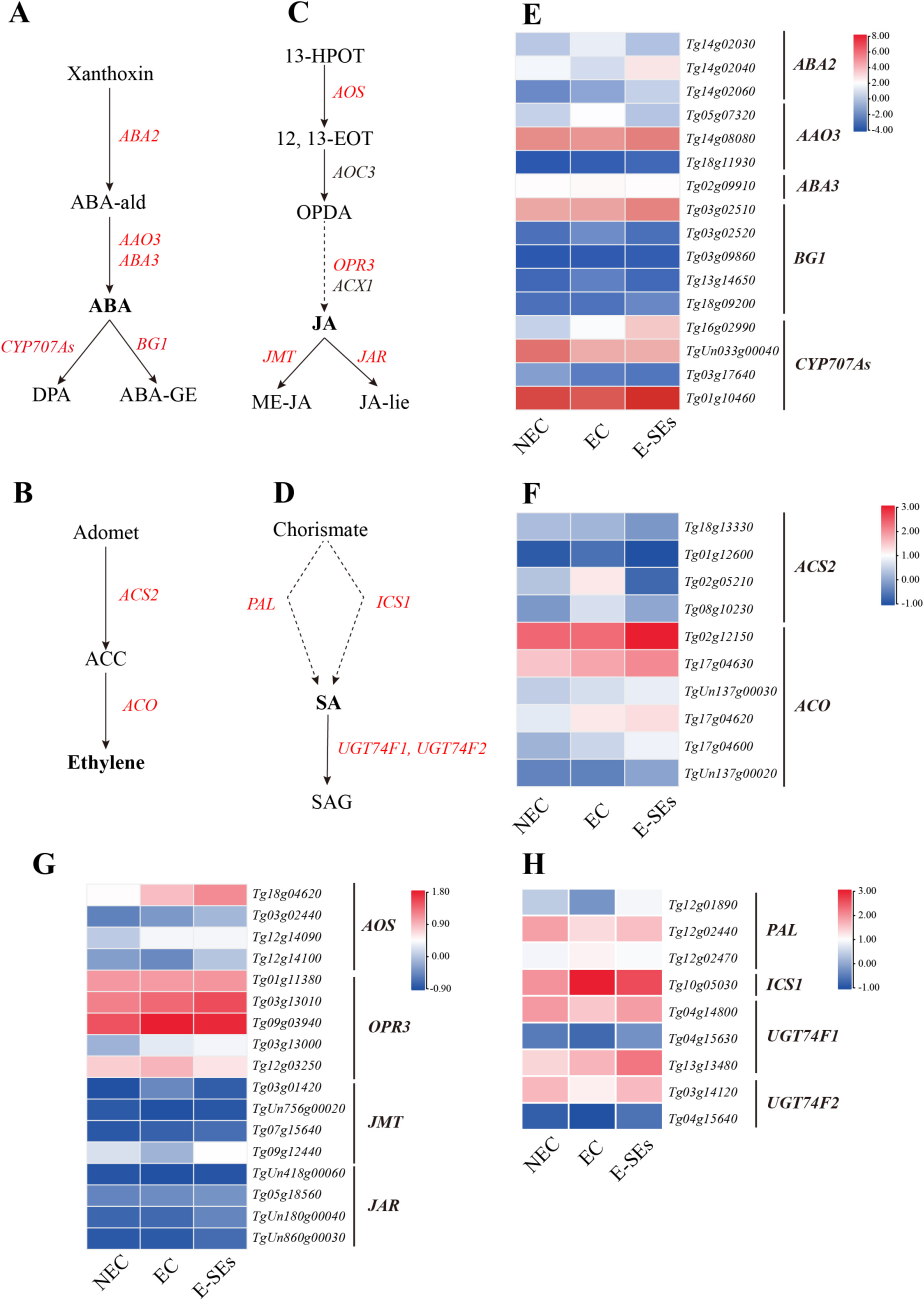
Dynamic changes in the expression of genes involved in ABA, ETH, JA and SA biosynthetic and metabolic pathways at different stages of SE in teak. **(A–D)**, Biosynthetic pathway of **(A)** ABA, **(B)** ETH, **(C)** JA, **(D)** SA. Substrates and products are denoted by plain text; genes encoding enzymes are denoted by italics. Red indicates the presence of homologous genes, black represents genes for which homology has not been detected. The detailed ABA and ETH biosynthetic and metabolic pathways were described in previous reports (Urano et al., 2017). The detailed JA biosynthetic and metabolic pathways were described in previous reports (Wasternack and Hause, 2013). The detailed SA biosynthetic and metabolic pathways were described in previous reports (Dempsey et al., 2011). **(E–H)**, Expression of genes involved in the **(E)** ABA, **(F)** ETH, **(G)** JA, **(H)** SA biosynthetic and metabolic pathways at different stages of SE. The heatmap shows the relative transcript levels of selected synthetic and metabolic genes. The color scale corresponds to the average log_10_(FPKM+0.1). *ABA2*, Short-chain alcohol dehydrogenase; *AAO3*, Abscisic aldehyde oxidase; *ABA3*, Molybdenum co-factor (MoCo) sulfurase; *BG1*, β-1,3-Glucanase 1. CYP707As, ABA 8’-hydroxylase activity; *ACS2*, ACC synthase; *ACO*, ACC oxidase; 13-HPOT, 13(S)-hydroperoxylinolenic acid; 12, 13-EOT, 12,13(S)-epoxylinolenic acid; JA-lle, jasmonoyl-L-Isoleucine; *AOS*, allene oxide synthase; *AOC*, allene oxide cyclase; *JAR1*, jasmonate-resistant1; *JAZ*, jasmonate ZIM-domain; SAG, Salicylic acid 2-O-β-glucoside; PAL, phenylalanine ammonia-lyase; *ICS1*, Isochorismate synthase1; UGT74F1/2, UDP-glycosyltransferase.

The role of ABA in plant embryo maturation has been extensively documented. Drawing upon previous studies (Urano et al., 2017), we investigated the ABA biosynthetic pathway. *ABA2* (*Tg14g02030*, *Tg14g02040*, and *Tg14g02060*) exhibited increased expression levels during the EC or E-SEs stages. The expression level of the key gene involved in ABA biosynthesis, *AAO3* (*Tg05g07320*), was elevated during the EC stage but decreased in the E-SEs stage (>2-fold) (**Figures 7A, E**). This finding aligns with the pattern of endogenous ABA accumulation during teak SE induction, contrasting with changes in the ABA precursor abscisic aldehyde (ABA-ald) (**Figure 4**). Furthermore, *ABA3* was not differentially expressed. These findings suggest that high levels of ABA play a crucial role in E-SEs induction in teak. In our analysis of ABA metabolic genes, the gene encoding the ABA metabolic enzyme *BG1* did not exhibit differential expression or low expression (FPKM < 1) during this process, and ABA-glucosyl ester (ABA-GE) was not detected. The expression of *CYP707As* exhibited distinct patterns (**Figure 7E**). For instance, *Tg16g02990* exhibited increased expression levels during the SE induction, whereas *TgUn033g00040* exhibited decreased expression levels.

ETH has been identified as a crucial factor in SE in *Arabidopsis* and soybean (Zheng et al., 2013). In this study, the expression of the *ACS2* gene (*Tg02g05210*, *Tg08g10230*), which encodes a key enzyme that catalyzes ethylene precursor biosynthesis, was found to increase during the EC stages, and its expression level decreased during the E-SEs stages (**Figures 7B, F**), which is consistent with the accumulation pattern of endogenous ACC during SE induction (**Figure 4**). Furthermore, the expression levels of the key ethylene biosynthesis gene *ACO* (*Tg02g12150*, *Tg17g04630*, *TgUn137g00030*, *Tg17g04620*, and *Tg17g04600*) showed sustained increases during the SE induction stages (>2-fold) (**Figures 7B, F**). These results indicate that ETH are continuously synthesized during SE induction.

JA plays a significant role in plant callus formation (Toro et al., 2003; Krajnčič et al., 2006). We analyzed the expression of genes involved in JA biosynthesis and catabolism in teak. Our findings indicated that during SE induction in teak, the expression of the JA synthetic gene *OPR3* (*Tg03g13000*, *Tg09g03940*, *Tg03g13010*, and *Tg12g03250*) increased, with *Tg09g03940* and *Tg12g03250* peaking in the EC stage, *Tg03g13000* and *Tg03g13010* showing the highest expression in the E-SEs stages (**Figures 7C, G**). This explains the increase in JA at the EC stage and the decrease in OPDA at the E-SEs stage. The JA metabolic gene *JMT* (*Tg09g12440*) exhibited decreased expression at the EC stage and increased expression at the E-SEs stages, consistent with the pattern of its product methylester-JA (ME-JA) changes. Interestingly, the gene encoding the JA metabolic enzyme gene *JAR* did not exhibit differential expression or low expression (FPKM < 1) during this process, and JA-lie was not detected (**Figure 7G**). These results suggest the involvement of the JA degradation pathway in teak globular and heart-shaped embryo formation.

Next, SA synthesis and metabolism were analyzed. SA synthesis involves two distinct pathways, cytosolic synthesis and chloroplastic synthesis (Métraux, 2002; Chen et al., 2009). We observed that the key enzyme gene *PAL* (*Tg12g01890*, *Tg12g02440*) in the cytosolic SA synthetic pathway exhibited decreased expression at the EC stage and increased expression at the E-SEs stage (**Figure 7D**), in accordance with the changes in endogenous SA concentrations during SE induction (**Figure 4**). Conversely, the expression of the key enzyme *ICS1* (*Tg10g05030*) in the chloroplastic pathway of SA synthesis showed opposite changes (**Figure 7H**). These results indicate the significant role of the cytosolic SA synthesis pathway during SE induction in teak. Moreover, two genes related to SA metabolism, *UGT74F1* (*Tg04g14800*, *Tg13g13480*) and *UGT74F2* (*Tg03g14120*), were differentially expressed. Notably, *Tg13g13480* showed a slow upregulation trend during the E-SE stages. On the other hand, *Tg04g14800* and *Tg03g14120* was significantly downregulated during EC and significantly upregulated during E-SEs (**Figure 7H**), which is consistent with the trend in the expression of the SA glucose conjugate (SAG) product (**Figure 4**). It can be speculated that SA plays a crucial role in teak globular and heart-shaped embryos formation through metabolic pathways.

### 3.6 The expression patterns of signal transduction-related genes during SE induction in teak

**Figure 8:**
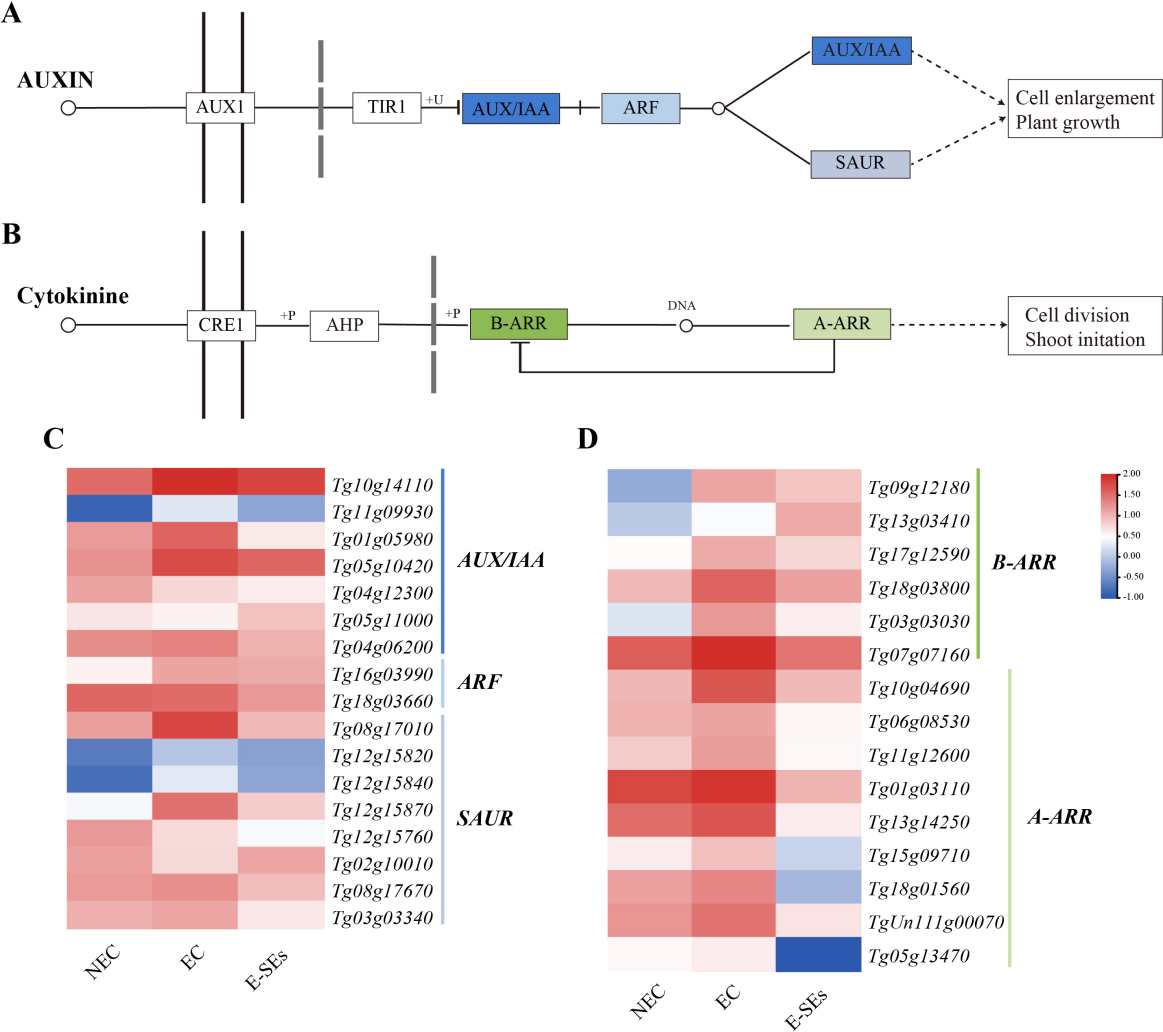
Enrichment analysis of DEGs related to plant hormone signal transduction during SE induction in teak. Genes related to the **(A, C)** auxin and **(B, D)** CTK signaling pathways and their expression patterns during SE induction in teak. The color scale corresponds to the average log10 (FPKM+0.1).

Plant hormone signaling plays a crucial role in SE (Su and Zhang, 2014; Elhiti and Stasolla, 2022). KEGG enrichment analysis of DEGs associated with the teak SE induction process suggested significant enrichment of the IAA and CTK signal transduction (**Figure 8**) (**Supplementary Table 4**). This includes key components such as ARF transcriptional repressor of auxin/indole-3-acetic acid (AUX/IAA), auxin response factor (ARF), small auxin-up RNA (SAUR), type-B response regulator (B-ARR), and type-A response regulator (A-ARR). In the auxin signaling pathway, most genes *AUX/IAA* (five genes, *Tg10g14110*, *Tg11g09930*, *Tg01g05980*, *Tg05g10420*, and *Tg04g06200*) and *SAUR* (five genes, *Tg08g17010*, *Tg12g15840*, *Tg12g15870*, *Tg08g17670*, and *Tg03g03340*) exhibited increased expression at the EC stage and decreased expression at the E-SEs stage (**Figure 8**). ARFs are the key transcription factors controlling auxin-responsive gene expression (Leyser, 2018). In teak, two differentially expressed *ARFs* were identified: *Tg16g03990* showed a >2-fold increase in the EC stage, while *Tg18g03660* showed a gradual decrease compared to that in the NEC stage. In the E-SEs stage, both genes displayed downregulated expression (**Figure 8C**). These findings suggest that during the transition from the NEC to EC stages, *Tg16g03990* plays a critical role in IAA signal transduction.

CTK signal transduction is thought to play a role in the early globular stage of SE in *Arabidopsis* (Müller and Sheen, 2008). In the CTK signaling pathway, genes such as *B-ARR* (five genes, *Tg09g12180*, *Tg17g12590*, *Tg18g03800*, *Tg03g03030*, *Tg07g07160*) and all *A-ARR* (nine genes) were upregulated during the EC stage and downregulated during the E-SEs stage (**Figure 8D**). We speculate that the downregulation of *B-ARR* at the E-SEs stage may be due to inhibition by the high expression of *A-ARR* in the EC stage. These results demonstrate the significant involvement of the auxin and cytokinin signaling pathways in teak somatic embryo development.

**Figure 9:**
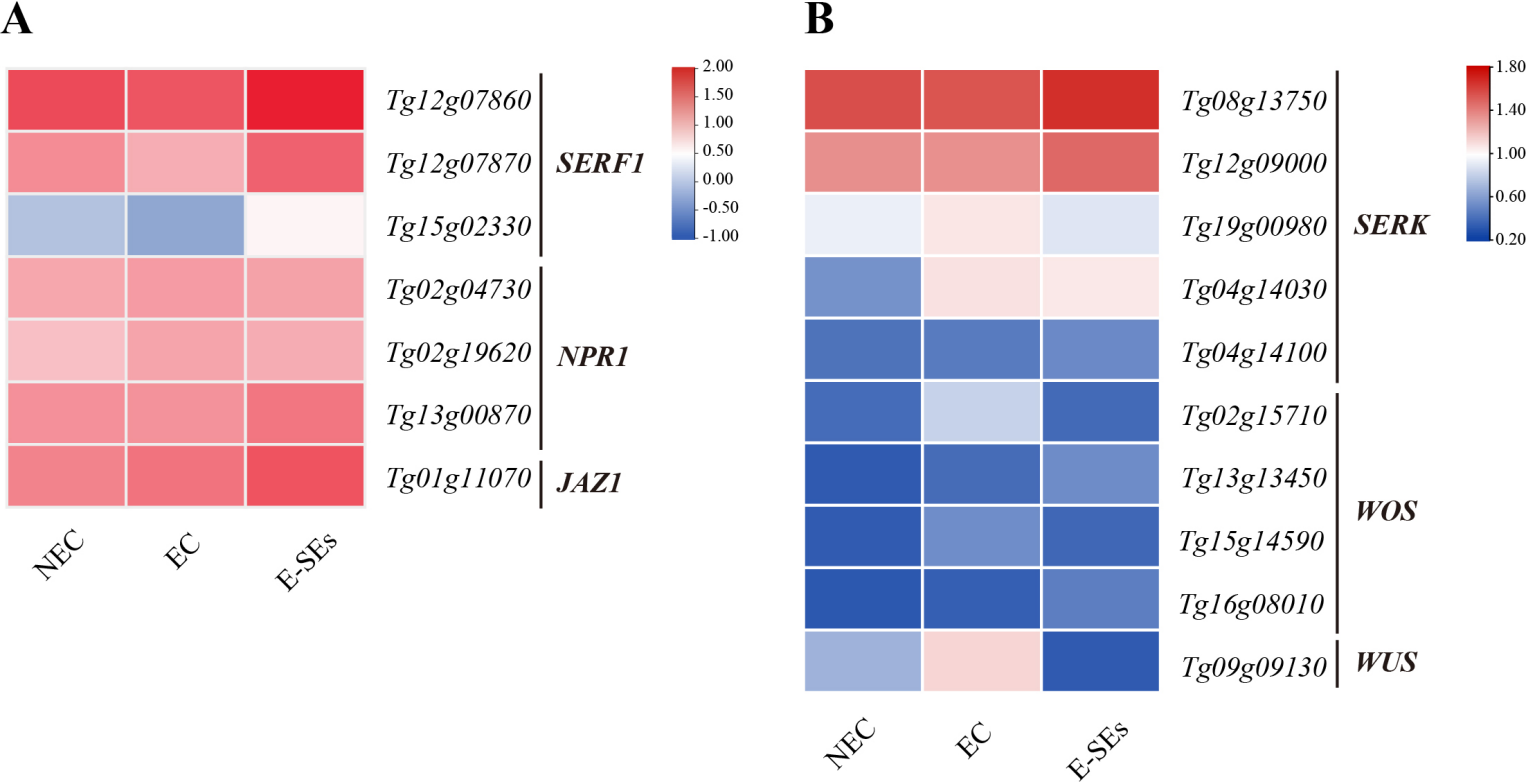
Expression patterns involved in the **(A)** ETH, JA, and SA signal transduction genes and **(B)** SE-related genes at different stages of SE. The heatmap shows the relative transcript levels of selected signal transduction genes. The color scale corresponds to the average log10 (FPKM+2).

In addition, we selected DEGs involved in other hormonal signal transduction pathways (**Supplementary Figure 8**) (**Supplementary Table 4**). Recent reports have shown that ABA mediates SE in *Arabidopsis* through the transcription factors ABI3 and ABI4 (Chen et al., 2021). In the different SE stages induced in teak, we selected one *ABI3* homologous genes (*Tg14g10080*). Interesting, there was no detectable expression of *Tg14g10080* transcript levels during the process of SE induction. In *Medicago truncatula*, the ethylene response factor somatic embryo related factor 1 (MtSERF1) is involved in SE (Mantiri et al., 2008). In teak, we identified three *MtSERF1* homologues, all of which showed a gradual decrease in transcript levels during the EC stage and a significant increase during the E-SEs stage, peaking at this point (**Figure 9A**). Furthermore, we selected *NPR1* and *JAZ*, which are related to SA and JA signal transduction and were not differentially expressed during the induction of somatic embryogenesis in teak (**Figure 9A**).

### 3.7 Expression of SE molecular marker genes during SE induction in teak

It is crucial to identify reliable molecular marker genes for distinguishing NEC from EC and SE. Several genes have been reported to be molecular markers of SE, such as somatic embryogenesis receptor kinase (SERK), Wuschel (WUS), and WUS-homeobox (WOX) (Li et al., 2011; Raúl et al., 2019). In our study, we selected the *SERK*, *WOX*, and *WUS* genes for investigation (**Supplementary Table 5**). Among the four identified *WOS* genes, the expression levels of *Tg02g15710* and *Tg15g14590* were highest during the EC stage (**Figure 9B**). Conversely, the expression levels of the other *WOS* genes, *Tg13g13450* and *Tg16g08010*, continuously increased, peaking during the E-SEs stage of SE induction (**Figure 9B**). Compared with those in the NEC and EC stages, the expression of one *WUS* gene (*Tg09g09130*) in the E-SEs stage significantly decreased. The expression of three *SERK* genes (*Tg19g00980*, *Tg04g14030*, and *Tg04g1410*) significantly increased, with that of *Tg04g1410* peaking during the E-SEs stage. Additionally, the expression of genes such as *Tg08g13750* and *Tg12g09000* gradually decreased during the EC stage and peaked during the E-SEs stage (**Figure 9B**). Our findings suggest spatial and temporal differences in the expression of distinct *SERK* genes during teak somatic embryogenesis induction.

### 3.8 Validation of gene expression using RT-qPCR

**Figure 10:**
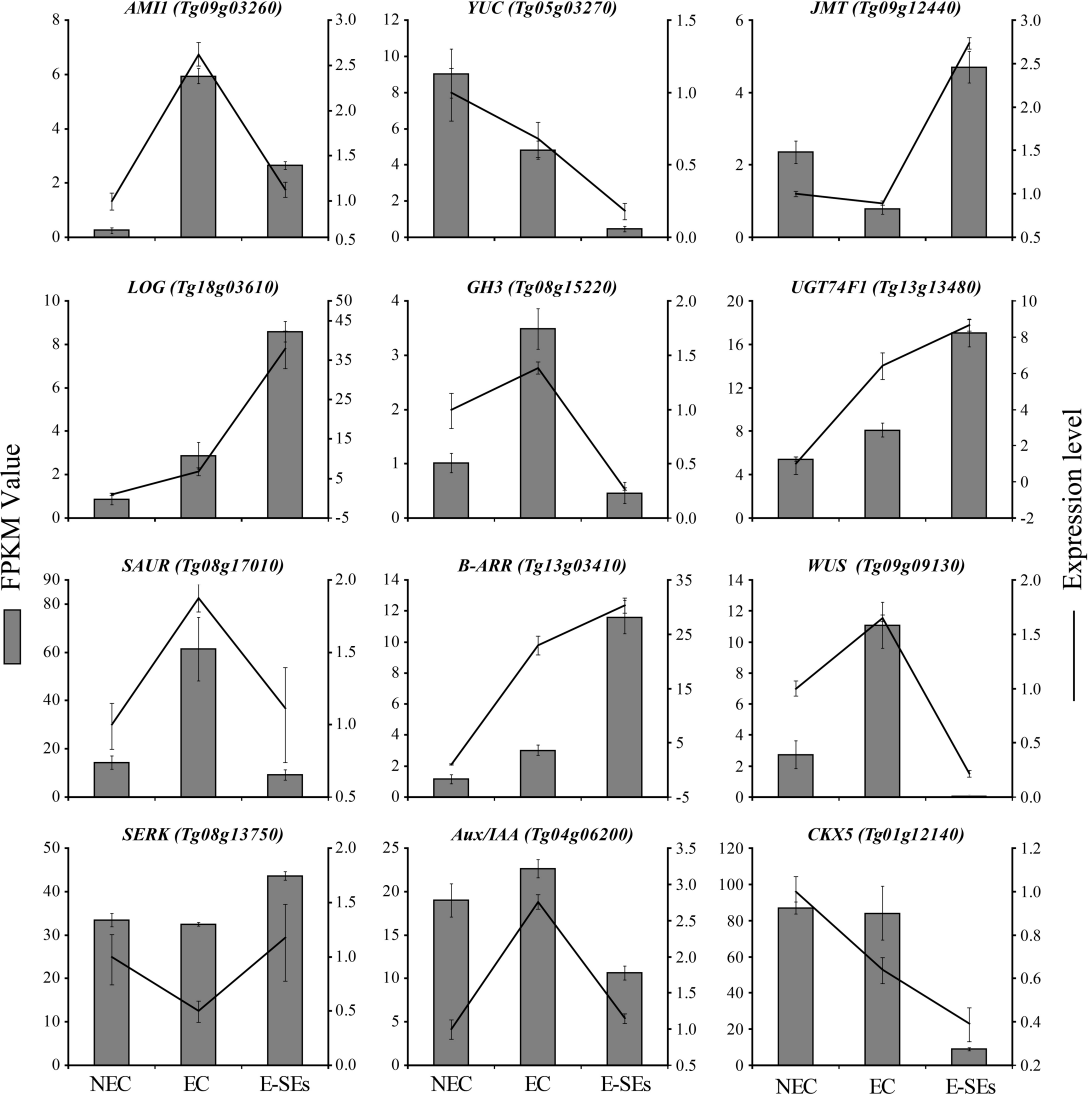
Comparison of expression profiles of twelve representative genes as measured by RNA-seq and RT-qPCR. Data are means ± SD (n =3). Error bars represent SD.

To validate the precision and repeatability of the RNA-Seq findings, twelve genes were chosen for RT-qPCR analysis. Ten genes including *AMI1* (*Tg09g03260*), *YUC* (*Tg05g03270*), *JMT* (*Tg09g12440*), *LOG* (*Tg18g03610*), *CKX5* (*Tg01g12140*), *GH3* (*Tg08g15220*), *UGT74F1* (*Tg13g13480*), *Aux/IAA* (*Tg04g06200*), *SAUR* (*Tg08g17010*), *B-ARR* (*Tg13g03410*), are related to plant hormones synthesis, metabolism, signal transduction. Two genes, including *WUS* (*Tg09g09130*) and *SERK* (*Tg08g13750*) are SE molecular marker genes during SE induction in teak (**Figure 10**). To 10 out of 12 genes, the genes expression patterns detemined by RT-qPCR were consistent with RNA-Seq results. Exceptions were observed in two genes, *SAUR* and *Aux/IAA*, which exhibited minor variation during the E-SEs stages.

## 4 Discussion

Plants can undergo totipotent growth, leading to the formation of complete plants through organogenesis or SE (Zhang et al., 2021). The organogenesis pathway has been extensively studied, utilizing mature and juvenile stems (Gupta et al., 1980), shoot tips (Bonga and Durzan, 1987), buds (Wirjodarmodjo et al., 1988), and axillary buds (Apavatjrut et al., 1990) for tissue culture systems in teak. SE plays a critical role in plant tissue culture regeneration, and elucidating its mechanisms is of paramount importance. However, SE is limited by genotype and plant growth regulators, posing significant challenges, particularly in woody plants such as teak.

Supplementation with exogenous plant hormones stimulates changes in the biosynthesis, transport, and metabolism of endogenous hormones within cells, leading to alterations in the intracellular concentrations of endogenous hormones. This subsequently affects gene expression regulation within cells, ultimately conferring embryogenic competence to somatic cells (Gulzar et al., 2020; Ding, 2017). Plant regeneration via SE can occur through direct induction, indirect induction, or a combination, depending on the tissue culture conditions and the nature of the explant (Paul et al., 2011; Loyola-Vargas, 2016). The successful establishment of both types of SE has been demonstrated in various species, including *Digitalis lanata* (Bhusare et al., 2020), *Camellia oleifera* (Zhang et al., 2021), and *Murraya koenigii* (Paul et al., 2011). In our study, we successfully induced early-stage somatic embryos, such as globular and heart-shaped embryos, in teak via an indirect induction approach on MS medium supplemented with TDZ, consistent with the method reported by Akram (Akram and Aftab, 2016). Distinct morphological characteristics were evident between NEC and EC during SE induction. Scanning electron microscopy observations of *Liriodendron* hybrid SEs revealed that the cells in the EC were spherical and uniform in size, predominantly exhibiting embryogenic cell clusters. In contrast, cells in the NEC exhibited irregular shapes and sizes. Similarly, our study demonstrated the presence of multiple embryogenic cell clusters within EC, suggesting that the formation of embryogenic cell clusters is essential for EC development (**Figure 1**) (Zhang, 2007; Jiang, 2010). However, we did not succeed in inducing torpedo embryos, cotyledon embryos and complete plants in our experiments, possibly because of the use of a single exogenous induction factor. Our forthcoming investigation will concentrate on assessing combinations of various hormones.

It is widely acknowledged that auxin and CTK play crucial roles in the regulation of cell division and differentiation, serving as key factors in triggering embryogenesis (Loyola-Vargas, 2016; Shi et al., 2020). Auxin is considered a central regulatory factor in SE, possibly due to the establishment of an auxin gradient during embryogenic induction (Yang et al., 2012). In *Hippeastrum* ‘Bangkok Rose’, the concentration of IAA was found to be lowest on day 0 of embryogenic induction, significantly increasing during the induction period and peaks on day 20 (Zeng et al., 2023). Moreover, in garlic, the concentration of IAA showed an initial increase followed by a decreasing trend during the embryo induction period, reaching a peak in the EC phase. In contrast, in our study, the highest IAA concentration was observed during the NEC phase and was undetectable during the EC and E-SEs periods (**Figure 4A**). This observation is consistent with the trend documented by Ayil, who reported a progressive decrease in IAA levels during SE induction in *Coffea canephora* (Ayil-Gutiérrez et al., 2013). However, the levels of the IAA biosynthesis-related gene *AMI1*, the *Tg09g03260* gene exhibited its maximum expression during the EC phase, while the expression of *Tg07g01540* reached its peak during the E-SEs phase. This provides a plausible explanation for the continuous reduction of its substrate IAM (**Figure 4A**). In addition, metabolic pathway genes *DAO1/2*, *GH3s* and signal transduction genes increased during EC and E-SEs (**Figure 5**). We speculate that the continuous synthesis of IAA and its rapid degradation through metabolic and signal transduction pathways are involved in SE in teak. The *YUC* gene encoding flavin monooxygenase is a key enzyme in auxin biosynthesis that can lead to an increase in the levels of the endogenous hormone IAA (Karami et al., 2023). Our research disclosed that one differentially expressed *YUC* gene presented a notable decrease, whereas the remaining genes manifested non-differential expression or low expression levels. Additionally,the intermediates IPA and IAN involved in auxin biosynthesis were also not detected (**Figure 4**). We speculate that IAA biosynthesis during SE induction of teak occurs predominantly through the IAM pathway.

Endogenous and exogenous CTK mutually regulate each other to achieve an internal balance, thereby initiating cell differentiation and redifferentiation (Ding, 2017). CTKs are well-known inducers of callus formation and are maintained at high levels in maize globular embryos (Ding et al., 2020). Conversely, in our study, the levels of IP decreased continuously during SE induction (**Figure 4**), while the levels of substances such as cZ and tZ and the transcription of genes involved in cZ synthesis did not significantly change (**Figure 6**). We speculate that exogenous TDZ may provide cytokinin-related functions for E-SEs formation. The mutual regulation of endogenous and exogenous cytokinins to achieve internal balance plays an important role in callus formation (Lv et al., 2023). In our study, the biosynthesis-related gene *LOG*, metabolic gene *CKXs*, and signal transduction genes *B-ARR*, and *A-ARR* exhibited varying peak expression levels across distinct phases of SE initiation, lacking a discernible trend (**Figure 6**). We posit that there is a complex regulatory network between exogenous cytokinin TDZ, endogenous cytokinin IP biosynthesis, metabolism, and transduction that requires further confirmation.

ABA plays a crucial role in the embryo development and maturation of zygotic embryos and SE (Vahdati et al., 2008; Tian and Brown, 2000). During the process of SE in switchgrass, carrot, sugarcane, grape, and melon, EC displays higher levels of endogenous ABA than does NEC (Su et al., 2013). Elevated endogenous ABA concentrations are associated with cellular reprogramming and the acquisition of embryogenic characteristics and play a significant regulatory role in SE (Jiménez, 2005). In *Arabidopsis*, fluridone, an inhibitor of *de novo* ABA biosynthesis, inhibits the transition of EC to somatic embryos (Kikuchi et al., 2006). Consistently, this study revealed a significant increase in ABA content during the EC stage, followed by a decrease during the transition to E-SEs (**Figure 4**). The expression patterns of genes encoding the ABA biosynthesis-related gene *AAO3* (*Tg05g07320*) are correlated with changes in endogenous ABA levels, suggesting that these genes play critical roles as key genes in ABA synthesis during SE induction in teak (**Figures 7A, E**). These results highlight the essential requirement of ABA during SE induction in teak; ABA is synthesized during EC and is involved in the induction of E-SEs. ABA induces SE by modulating intracellular local auxin biosignaling (Su et al., 2013). Although the ABA signal transduction-related genes were not detected in our study, the *CYP707As* metabolic gene exhibited distinct expression patterns, leading to the hypothesis that ABA induces SE through interacting with other hormones.

Previous research has indicated that ETH is a crucial hormone involved in initiating and developing during SE (Hatanaka et al., 1995; El Meskaoui et al., 2000; Mantiri et al., 2008), participating in various stages of SE (Mauri and Manzanera, 2011). ETH levels increase during the EC stage in *Medicago truncatula* callus (Huang et al., 2001). Similarly, the precursor of ETH, ACC, exhibited the highest levels during the EC stage (**Figure 4**), with continuous upregulation of the key gene *ACO* involved in ETH synthesis (**Figure 7F**). Moreover, the expression of the ethylene response factor gene *SERF1* (*Tg12g07860*, *Tg12g07870*, and *Tg15g02330*) was the lowest during the EC stage, suggesting ethylene accumulation at this stage. *MtSERF1* appears to be essential for SE in *Medicago truncatula* and is strongly expressed in globular somatic embryos (Mantiri et al., 2008). Consistently, in our study, all *SERF1* genes exhibited the highest expression levels at the E-SEs stage, as globular embryos are in the E-SEs phase. These findings suggest that high *SERF1* expression during E-SEs is essential for GE formation. In *Arabidopsis*, ETH downregulates auxin biosynthesis by inhibiting *YUC* expression, disrupting local auxin distribution (Bai et al., 2013). In our studys, the expression of the *YUC* gene is not significantly different or is downregulated during SE induction in teak. We speculate that ETH inhibits the expression of these genes, leading to IAA synthesis via the IAM pathway.

JA plays a significant role in the embryogenic process of plant somatic cells, exerting distinct regulatory effects depending on its concentration (Cheng et al., 2017; Ge, 2016). Treatment with JA has been shown to stimulate the production of abundant somatic embryos in cotton globular embryos (Ge, 2016). Notably, JA showed peak expression levels in the EC and the expression of the JA synthetic gene *OPR3* increased during SE induction. We suggest that elevated levels of JA promote the transformation of teak EC into globular and heart-shaped embryos. It has been reported that the addition of ME-JA delays the transition of embryogenic cell clusters to torpedo-stage embryos and significantly inhibits the regenerative capacity of somatic embryos (Ruduś et al., 2006). In our study, JA primarily produces ME-JA through metabolism, the level of which decreases along with *JMT* (*Tg09g12440*) during EC and increases in expression during E-SEs (**Figures 7C, G**). We speculate that high concentrations of ME-JA suppress the transition from globular and heart-shaped embryos to torpedo embryos in teak.

The impact of SA on SE induction is species- or genotype-dependent. In *C. delgadii* (Grzyb et al., 2018), an increase in the SA concentration in the culture medium results in a decrease in the number of somatic embryos, with complete inhibition of somatic embryogenesis observed at 125 μM. In *Coffea arabica* suspension culture, picomolar concentrations of SA led to a two fold increase in cell growth and SE efficiency, while a concentration of 1 μM inhibited these processes (Quiroz-Figueroa et al., 2001). Similarly, in our study, during the EC stage, the concentration of JA and the expression levels of the key synthetic gene *PAL* (*Tg12g01890*, *Tg12g02440*) were the lowest, and low concentrations of SA promoted the transition of EC to E-SEs (**Figures 7D, H**).

**Figure 11:**
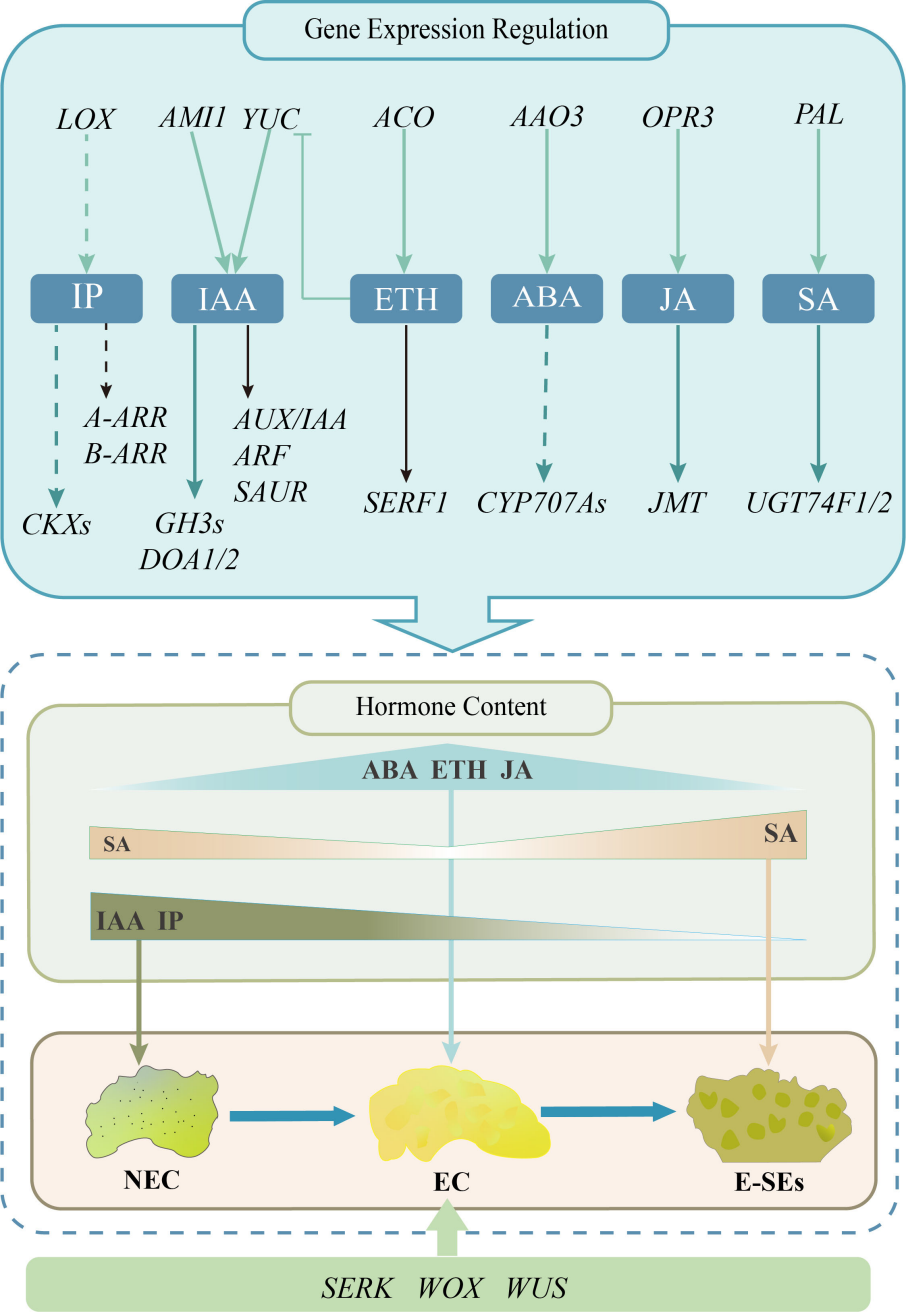
Proposed working model for the expression profile of plant hormones during early somatic embryo formation in teak.The pastel green arrows signify genes linked to the stimulation of hormone biosynthesis, while the deep green arrows represent genes pertinent to hormone metabolism. Genes related to hormone signal transduction are denoted by the black arrows. The obstructed arrows are suggestive of suppressed expression, while the dotted arrow infers the requirement for additional validation.

In conclusion, our study revealed plant hormone expression profiles during different SE stages induced in teak. During the transition from NEC to EC, the concentrations of the endogenous hormones IAA, IP, and SA decrease. During the process of EC transformation into E-SEs, the concentrations of the endogenous hormones ABA, ETH, and JA decrease. By integrating transcriptomic data, we elucidated the associations between genes related to biosynthesis, metabolism, signal transduction, and endogenous hormones during SE induction in teak. Along with SE induction, key regulators, including *SEKR* (five genes), *WOX* (four genes), and *WUS* (one gene), were differentially expressed and regulated the induction of teak somatic embryos (**Figure 11**).

## 5 Conclusions

In summary, early-stage somatic embryos, including globular and heart-shaped embryos, were induced from teak callus in our study. We integrated hormonal content and transcriptomic data to reveal the expression profiles of genes involved in plant hormones such as IAA, iP, ABA, ETH, SA, JA biological synthesis, metabolism, and signal transduction processes, as well as representative molecular marker genes, indicating their possible roles in teak SE. These data provide new insights for future functional studies as a means of studying the molecular mechanisms involved in SE.

## Data Availability

The datasets presented in this study can be found in online repositories. The names of the repository/repositories and accession number(s) can be found below: https://www.ncbi.nlm.nih.gov/, PRJNA1106626.
