# Supplementary material for: Analysis of the plant hormone expression profile during somatic embryogenesis induction in teak (Tectona grandis)
Source: Front Plant Sci. 2024 Oct 7;15:1429575. doi: 10.3389/fpls.2024.1429575 (PMC11494608; doi:10.3389/fpls.2024.1429575)
Supplement: Supplementary file 2 [file DataSheet2.zip › Supplementary Figure/Supplementary Figure 1.docx]

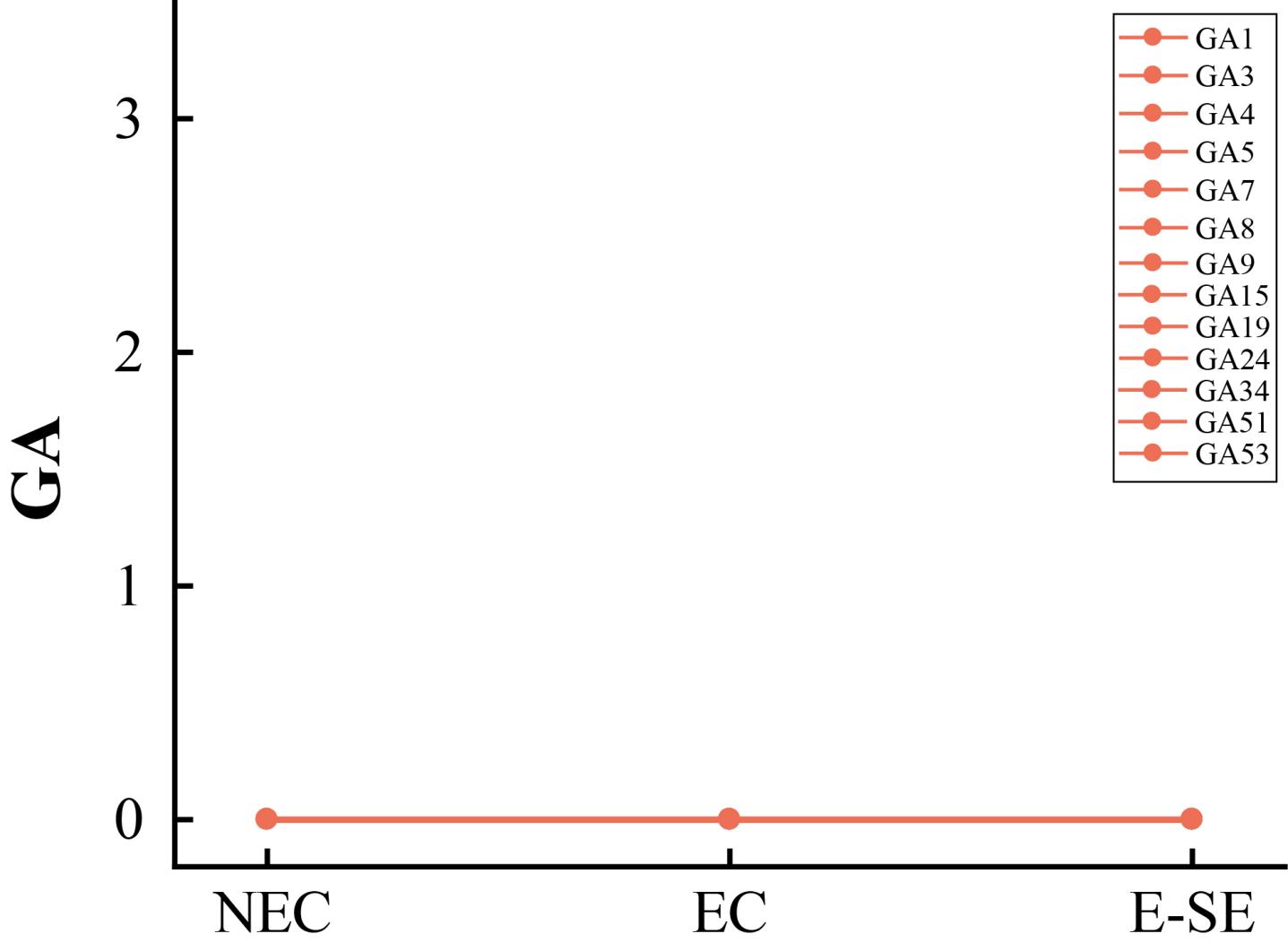


**Supplementary Figure 1.** Dynamic changes in the levels of GA. GA1, Gibberellin A1; GA3, Gibberellin A3; GA4, Gibberellin A4; GA5, Gibberellin A5; GA7, Gibberellin A7; GA8, Gibberellin A8; GA9, Gibberellin A9; GA15, Gibberellin A15; GA19, Gibberellin A19; GA24, Gibberellin A24; GA34, Gibberellin A34. GA51, Gibberellin A51; GA53, Gibberellin A53.
