# Supplementary material for: Analysis of the plant hormone expression profile during somatic embryogenesis induction in teak (Tectona grandis)
Source: Front Plant Sci. 2024 Oct 7;15:1429575. doi: 10.3389/fpls.2024.1429575 (PMC11494608; doi:10.3389/fpls.2024.1429575)
Supplement: Supplementary file 2 [file DataSheet2.zip › Supplementary Figure/Supplementary Figure 2.docx]

TAA1/TIR1/TAR2 (Identity = 51.4%)

YUC (Identity = 65.69%)

AMI1 (Identity = 35.76%)

GH3s (Identity = 52.4%)

DAO1/DAO2 (Identity = 65.97%)

CYP71A13 (Identity = 66.1%)

NIT1NIT2 (Identity = 46.02%)

**Supplementary Figure 2.** Multi-sequence alignment of amino acid sequences of homologous genes involved in IAA biosynthesis and metabolism. Black highlights indicate homology levels greater than or equal to 100%, red indicates homology levels greater than or equal to 75%, and blue indicates homology levels greater than or equal to 50%.
