# Supplementary material for: Analysis of the plant hormone expression profile during somatic embryogenesis induction in teak (Tectona grandis)
Source: Front Plant Sci. 2024 Oct 7;15:1429575. doi: 10.3389/fpls.2024.1429575 (PMC11494608; doi:10.3389/fpls.2024.1429575)
Supplement: Supplementary file 2 [file DataSheet2.zip › Supplementary Figure/Supplementary Figure 5.docx]

ACS2 (Identity = 63.33%)

ACO (Identity = 84.5%)

**Supplementary Figure 5.** Multi-sequence alignment of amino acid sequences of homologous genes involved in ETH biosynthesis and metabolism. Black highlights indicate homology levels greater than or equal to 100%, red indicates homology levels greater than or equal to 75%, and blue indicates homology levels greater than or equal to 50%.
