# Supplementary material for: Analysis of the plant hormone expression profile during somatic embryogenesis induction in teak (Tectona grandis)
Source: Front Plant Sci. 2024 Oct 7;15:1429575. doi: 10.3389/fpls.2024.1429575 (PMC11494608; doi:10.3389/fpls.2024.1429575)
Supplement: Supplementary file 2 [file DataSheet2.zip › Supplementary Figure/Supplementary Figure 8.docx]

ABI3 (Identity = 38.37%)

JAZ1 (Identity = 37.93%)

NPR1 (Identity = 70.44%)

SERF1 (Identity = 39.31%)

**Supplementary Figure 8.** Multi-sequence alignment of amino acid sequences of homologous genes involved in signal transduction. Black highlights indicate homology levels greater than or equal to 100%, red indicates homology levels greater than or equal to 75%, and blue indicates homology levels greater than or equal to 50%.
